# Baseline survey of environmental parameters, radiation, and drip water hydrochemistry in Niah Caves (Sarawak, Malaysia)

**DOI:** 10.1007/s10661-024-13133-9

**Published:** 2024-09-19

**Authors:** Prasanna Mohan Viswanathan, Dominique Dodge-Wan

**Affiliations:** Department of Applied Sciences, Faculty of Engineering and Science, Curtin University Malaysia, CDT 250, 98009 Miri, Sarawak Malaysia

**Keywords:** Karst, Hydrochemistry, Radiation, Wind, Humidity

## Abstract

Relict caves in fenglin karst may typically have numerous entrances and openings. Hence, they host a variety of environments in which parameters such as light, airflow, humidity, and temperature may vary significantly over short distances. Similarly, drip water hydrochemistry, including isotopic values, may vary due to different contributions of various sources and residence time in the karst. This study investigated environmental parameters, including radiation, using hand-held instruments, along a transect within several major caves in the Niah karst of Sarawak (Malaysia). This has led to a baseline data set which showed an inverse relationship between humidity and temperature, gamma radiation levels that are about 25% of that in surrounding non-karst region, and high percentages of twilight zones in the studied caves. Airflow was found to be variable, with high values of 530 m^3^/s in Painted Cave and 122 m^2^/s in parts of Gan Kira passage, with flow towards the southeast at the time of the study. The hydrochemistry of the drip water and surface water was also analyzed and found to be dominantly Ca–Mg–Cl water type which indicates dissolution of minerals through water–rock interaction. In addition, the cave environment, particularly air temperature, humidity, and ventilation, also influences the drip water composition and isotopic values. Three different origins (precipitation, evaporation, and paleo-recharge) of drip water have been identified through the *δ*^18^O-*δ*^2^H diagram. Enriched isotopic values were observed in the cave entrance due to increased evaporation caused by lower humidity and higher air temperature. Factor analysis identifies the key geochemical processes responsible for the drip water chemistry. The outcome of this study provides the first baseline environmental data for the Niah caves, which could support future initiatives for sustainable management of this famous archeological site in southeast Asia.

## Introduction

Niah caves are well known for their rich archeological content and traditional edible birds’ nest harvesting industry. Like many tropical caves in small fenglin karst towers, the Niah caves are typically well connected to the outside environment due to multiple large entrances and numerous ceiling skylights (Dodge-Wan, 2023). Connectivity with the outside swamp forest is critical for the fauna and flora that inhabit or visit various parts of the cave. The biodiversity includes, for example, a large population of swiftlets (Black-Nest, Mossy-Nest, and White-Bellied species) and bats, with over 30 bat species reported roosting in the caves (Anwarali Khan et al., 2008; Leh & Kheng, 2001; Lim & Cranbrook, 2014).

The entrance zone of the Painted Cave is the location of the first reported crayback stalagmite in Borneo (Lundberg & McFarlane, 2011) and hosts one of the world’s largest clusters of this type of cyanobacteria-influenced stalagmite (Dodge-Wan et al., 2012; Dodge-Wan & Deng, 2013). Other crayback-like stalagmites are also noted in other cave entrances at Niah (Dodge-Wan, 2023). The spatial and temporal variation in the geochemical composition of drip water in selected sites within Niah caves reveals the water–rock interaction is the primary control for drip water chemistry (Prasanna et al., 2014). It was also observed that the geochemistry of a cave stream is influenced by the dissolution of the host rock (i.e., cave wall along the passage) and the leaching of organic matter (Dodge-Wan et al., 2017).

Hasegawa et al. (2018) point out that there have been limited environmental studies in tropical caves worldwide, despite the impact environmental parameters have on stalagmite growth. Furthermore, environmental parameters are important for the fauna and flora in various parts of the cave. This study, which is the first of this kind, aims to establish baseline values for a number of environmental parameters at Niah, principally within the main caves.

Previous studies on drip waters in Niah Cave (Prasanna et al., 2014, 2016) were limited in terms of sample numbers and the chemical data, including isotopes. In this study, 12 drip water samples have been collected and analyzed, to cover all the main caves (TC, GC, GK, and PC) in the Niah cave area. In addition, cave environmental parameters have been compared with the drip water chemistry to depict the overall cave condition for the formation of speleothems through precipitation from drip water.

The aim of the study is to establish baseline environmental parameters against which future changes may be evaluated. This may be significant for better understanding the conditions under which speleothems develop, the impact of climate change and increasing visitor numbers, the preservation of cultural relicts, and the need to maintain the cave environment for fauna and flora therein.

## Study area

Niah is one of Sarawak’s key karst areas, the other two being Mulu (approximately 100 km east southeast of Miri) and Bau (approximately 28 km southwest of Kuching). The Niah karst is located 18 km inland from the South China Sea in the low-lying coastal area and is approximately 65 km southwest of Miri. The Subis Limestone Formation is an Oligocene to Miocene carbonate reef build-up, with facies interpreted to be from the inner and outer reef talus, reef rim, and lagoon (Saw et al., 2019). The limestones in which the caves formed have been eroded into steep-sided karst towers, or fenglin, within an area approximately 4 to 5 km in diameter. The karst area is surrounded by the older sandy Nyalau Formation and the younger Setap Shale Formation.

**Fig. 1:**
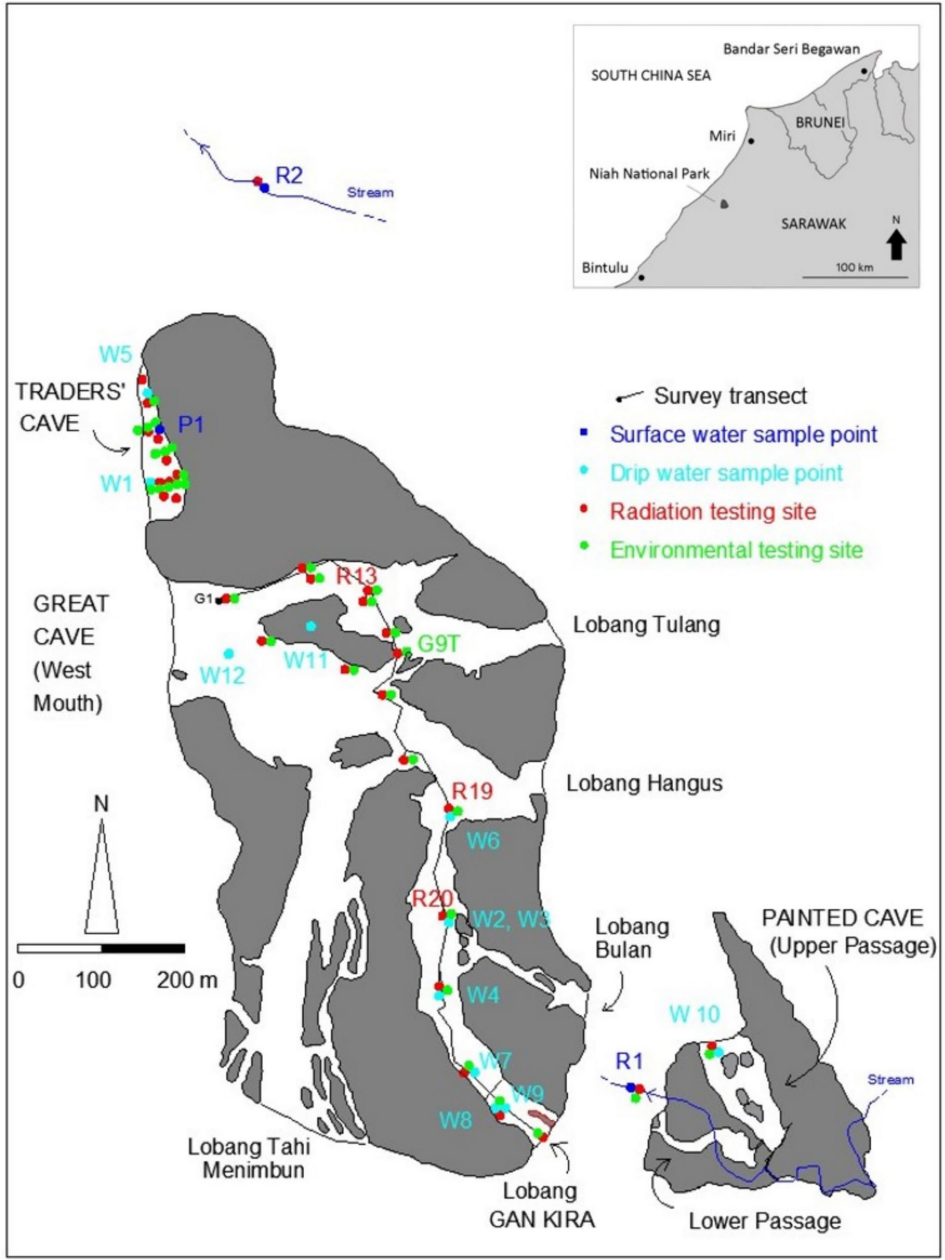
Map of Niah Great Cave and Painted Cave karst towers, showing cave names and entrance names with hydrochemistry sample points, radiation testing sites, and environmental testing sites as well as survey transect through Great Cave to Lobang Gan Kira. The surrounding area (not depicted) is occupied by forest. The inset map (top right) shows the location of Niah National Park in NW Sarawak

The area of interest of this research includes the three main tourist caves: Traders’ Cave (TC), Great Cave (GC), and Painted Cave (PC) shown in Fig. 1. TC and GC are within one of the main karst towers, while the PC is in an adjacent smaller tower (Dodge-Wan, 2018; Dodge-Wan et al., 2017). TC is a single large elongated passage over 150 m long, and open by erosion on the west side (Dodge-Wan, 2018). The GC is a very large and complex cave with numerous large entrances: West Mouth (Kuala Besar), Lobang Tulang, Lobang Hangus, Lobang Bulan, Lobang Gan Kira (GK), Lobang Tahi Menimbun (listed in clockwise order around the tower from West Mouth) (Hazebroek & Morshidi, 2000). There are also a number of ceiling skylights where erosion has opened the roof of the cave. The West Mouth is well known for its unique archeological content (Barker et al., 2002; Curnoe et al., 2018). More recently, excavations have focused on the Traders’ Cave (Chua, The Borneo Post 23 October 2018). All three caves are considered relict, being isolated above the regional water table which is only observed in other small epiphreatic passages (Dodge-Wan et al., 2017). The area is in the humid tropics with high humidity and temperature throughout the year. At sea level, the average temperature is 27 °C, and the average annual rainfall is around 2800 mm (Prasanna et al., 2016).

## Methodology

### Survey

All measurement stations for this study were surveyed and linked. The survey method consisted of traversing the route and measuring distance, azimuth, and slope between each survey station using a tripod-mounted Leica Disto D810 range finder. Additional measurements of cave width (left and right of station) and height (above and below station) were made at selected survey stations and used to map cave walls. The survey route through the Great Cave starts at a datum point selected at a walkway near the entrance to the archeological site, referred to here as “datum G1” and shown in Fig. 1. AutoCAD2014 software was used to accurately plot the data and compare it with pre-existing surveys. Previously published surveys of caves at Niah include Wilford (1964) and Hazebroek and Morshidi (2000) which is modified after Wilford (1964). More recent detailed published surveys include our authors’ previous surveys at Niah at the PC tower (Dodge-Wan et al., 2017) and at the TC (Dodge-Wan, 2018) which were incorporated here. Passage cross-sectional areas were based on detailed passage cross-sections using the same instrumentation. Cave floor surface areas in the map view were computed using AutoCAD.

### Environmental parameters

Environmental parameters (air temperature, airspeed, relative humidity, light) were measured using Sper Scientific Mini Environmental Quality Meter model 850070. The instrument provides the following accuracy: ambient temperature ± 1.2 °C, airspeed ± 3% m/s, relative humidity ± 8% (above 60% RH), and light intensity ± 5% lux (Sper Scientific, 2022). The detection level for airspeed is 0.4 m/s. For airspeed, the instrument was held in the direction of maximum cave wind at approximately 1.2 m above the cave floor. For lighting, it was pointed towards the nearest source of light, which might be a cave entrance or ceiling skylight depending on the location. The environmental testing sites are shown in Fig. 1.

The four parameters (air temperature, airspeed, relative humidity, and light) were recorded at each individual site at the same date and time. Readings were recorded on 25/3/2019 between 13:05 and 16:20 at Traders’ Cave, on 26/3/19 between 09:40 and 15:40 at Great Cave, and on 27/3/19 between 11:00 and 17:00 at Gan Kira and Painted Cave.

### Radiation

Radiation measurements consisted of separate gamma and beta flux values, both acquired with the Polimaster PM1405 instrument. For gamma readings (ambient dose equivalent rate in air or TGRD) the instrument was placed at 1 m above the ground surface on a tripod at the survey station, and allowed to stabilize till the statistical error level reached below 20%. The values are reported here in nGy/h. In addition, beta flux values in counts per second (CPS) for the detector of 7 cm^2^ were recorded by placing the instrument directly on the surface to be tested, with the back panel in the open position. The values represent “joint beta plus gamma radiation by beta-flux modification” (Polimaster, 2009) after stabilization to a statistical error level below 15%. The radiation testing sites are shown in Fig. 1.

### Drip water and surface water analysis

A total of 12 drip water samples were collected from different sites in the Niah caves complex during the end of the northeast monsoon (March 2019). This includes two samples (W1 and W5) from TC, three samples (W6, W11, and W12) from GC, six samples (W2–W4, W7–W9) from GK, and one sample (W10) from PC (Fig. 1). The water samples were mostly dripping either from fractures in cave ceiling or from stalactites. In addition, three surface water samples were collected and analyzed: one sample (R2) from the stream in the forest to the north of Great Cave tower, one sample from the stream flowing through Painted Cave tower (R1), and one sample from a small pool inside Traders’ Cave (P1) (Fig. 1). Temperature, pH, electrical conductivity (EC), total dissolved solids (TDS), and salinity were measured in situ using portable meters. For the drip water, large polyethylene buckets were used to collect the samples directly from the dripping spots, and a water scooper was used to collect the surface water samples. The collected samples were stored in polyethylene bottles and kept at a temperature of 4 °C for further elemental analysis. In the laboratory, all the samples were filtered using a 0.45-micron filter membrane. Cl^−^ and HCO_3_^−^ were analyzed using the titrimetry method (APHA, 1998). Ca^2+^, Mg^2+^, Na^+^, and K^+^ were analyzed with a Flame Atomic Absorption Spectrophotometer (PerkinElmer A Analyst 400). Calibration curves were created using standards prepared from stock solutions for the accuracy of the Atomic Absorption Spectrophotometer. Quality checking was carried out randomly by cross-checking the values with the standards to ensure the accuracy of the results. Nutrients such as SO_4_^2−^, NO_3_^−^, and NO_2_^−^ were analyzed using a UV visible spectrophotometer (DR2800) with Hack test kits (powder pillows) for NO_3_^−^ (cadmium reduction method 8196), and NO_2_^−^ (diazotization method 8507) and SO_4_^2−^ (sulfaVer 4 method 8051). Ionic charge balance error percentage was also calculated for total cation and total anion, and it was found that the error was within the acceptable range between 5 and 10% (Domenico & Schwartz, 1998). Oxygen and hydrogen isotopes for seven drip water samples were measured by using Isotopic Ratio Mass Spectrometer (IRMS). The WATEQ4F geochemical model (Ball & Nordstrom, 1992) was used to compute the saturation index (SI) for carbonate and sulfate minerals. Factor analysis was performed using SPSS software (version 17) to establish the ionic relationship, which controls geochemical processes in cave water (Nepolian et al., 2022; Rakesh et al., 2023).

## Results

### Survey

The Great Cave (GC) is a very large and complex cave, with passages commonly exceeding 40 m in width. There are numerous entrances and ceiling skylights. The West Mouth is over 110 m wide, and in the south portion, the ceiling is over 50 m high.

The new survey information obtained with modern instruments during this study, consists of a complete traverse through GC, via the normal visitor route to establish the location of the measuring stations. The trace of the route plots within cave voids is shown on Wilford’s map (1964). Some differences in the position of cave walls adjacent to the route can be accounted for by differences in surveying at different heights in complex cave passages.

The survey route through GC from G1 near the archeological site in West Mouth to the Gan Kira (GK) entrance is over 980 m long. Along the survey route within GC, there is an elevation increase of + 24.78 m (above datum at G1) with the highest surveyed ground within the cave located at the top of the sediment mound (station R13). The lowest point surveyed within GC is in GK (station R20) at − 14.41 m (below the datum at G1). The main section of GK (southeast entrance) was found to be at approximately − 13 m (below datum at G1). Within the GC central zone, there are several ceiling skylights. The highest one is over 100 m vertical distance above the floor (station R19).

### Air temperature

The statistical values for humidity, air temperature, and wind speed tested in this survey are provided in Table 1. Air temperature readings were taken at 13 locations within TC on 25 March 2019 in the afternoon, in Great Cave on 26 and 27 March 2019 between 9 am and 5 pm (17 measurements), and outside in the forest near PC (1 measurement) and in PC (1 measurement).

**Table 1:** Statistical values for the environmental parameters (humidity, air temperature, and wind speed) tested in this survey

Parameter	*n* (number of measurements)	Unit	Minimum	Maximum	Mean	Standard deviation
Humidity in caves	18	% RH	72.3	90.1	80.8	5.4
Humidity in forest	1	% RH	-	-	91.2	-
Air temperature in caves	18	° C	25.9	30.5	28.1	1.2
Air temperature in forest	1	° C	-	-	27.3	-
Wind speed in caves	6^1^	m/s	0.2	2.5	1.4	0.7

^1^the additional 11 wind speed measurements that are BDL were not included in the calculations

In TC during hot afternoon, the temperature values ranged from 28.4 to 29.8 °C. The highest values were measured at the open side of the cave in the northwest, adjacent to the forest. The lowest value was recorded behind a large stalagmite, close to the cave wall on the east side. The average temperature was 29.0 °C.

In GC a higher degree of variability was noted, with measurements on two consecutive days. The highest value was 30.5 °C in the north sector of the West Mouth which is exposed to direct sun. On both days, the lowest temperatures (< 27 °C) were recorded in the northwest to southeast trending GK passage, within 250 m of the GK entrance. The air temperature was 29.2 °C in PC on 27 March 2019 early afternoon, which is 2° warmer than in the adjacent forest on the same day within the same hour.

### Humidity

**Fig. 2:**
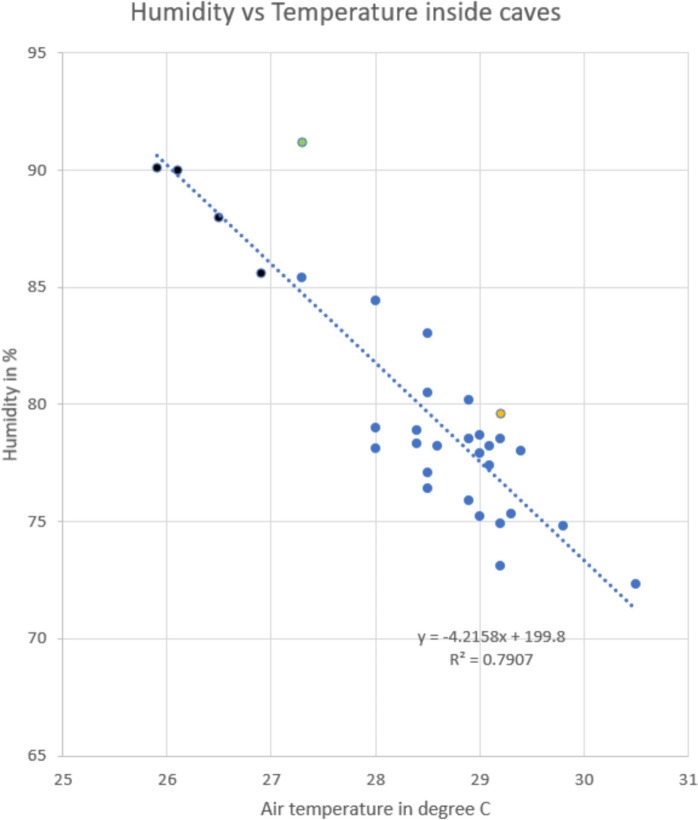
Correlation between humidity and air temperature with points in blue from TC and Great Cave, points in black from GK, point in yellow is inside PC, and outlier point in green is in the forest near PC

Thirty-two relative humidity (RH) measurements were made, at the same locations, day, and time as air temperature over the 3 days of survey in different parts of the cave system. The results indicate averages of 77.5% RH in TC and 80.9% RH in GC. In the GC central dark zone, the relative humidity ranged from 73 to 79%. Inside the caves, the highest humidity (between 85 and 90%) was recorded in GK. However, the humidity in the swampy forest between Niah tower and PC tower was even higher at 91.2% (Table 1). The humidity was found to show a strong inverse correlation (*R*^2^ = 0.79) with air temperature (Fig. 2).

### Wind

Attempts were made to measure airspeed (wind velocity) at 26 locations within the caves. At all but six locations, the airspeed was below the detection level. The locations where airspeed was measurable are described here below.

In GC, a wind velocity of 0.2 m/s was recorded at the top of the walkway junction (point G9T) with the wind blowing towards the east entrance of Lobang Tulang. In the south portion of West Mouth, a wind velocity of 1.8 m/s was recorded, blowing towards the southeast. In the entrance zone of the GK passage, the wind was blowing out of the cave (to the southeast) at a velocity of 1.1 to 1.2 m/s. The cross-sectional area of the passage (limited to the section with timber walkway where velocity was measured) is approximately 106 m^2^ which gives an airflow rate of 122 m^3^/s in Gan Kira.

The strongest wind was recorded at the entrance of PC, with a velocity of 2.5 m/s inwards towards the southeast at 2 pm on 27 March 2019. This cave is a “light through cave” with cave passage aligned in the northwest to southeast direction, and cave wind is frequently noticeable and strong in this location (Dodge-Wan, 2023; Dodge-Wan & Deng, 2013). The cross-sectional area of the passage (limited to the central zone) is approximately 212 m^2^ which gives an airflow rate of 530 m^3^/s in Painted Cave.

No clear relationship was obvious between wind speed, temperature, and humidity, for the six locations where the wind speed was above detection or considering the presence or absence of detectable airflow. Long-term simultaneous readings would be required to establish possible relationships.

### Light

Light intensity was measured at 34 locations, within the caves and at the time of measurement ranged from 1750 lx close to large cave openings to zero in the dark zone, in areas where there are no ceiling skylights. The testing locations are shown in Fig. 1 in relationship to the cave passage and entrances. The light intensity varies spatially depending on the exact configuration and orientation of the entrances and the presence of possible obstructions such as speleothem columns and boulders or bends in passage walls. It is also expected to vary with the time of day and the direction of incoming sunlight.

The survey shows that light intensities > 30 lx can occur over 150 m into the cave from very large openings like the West Mouth (over 100 m wide). Similar or higher values were observed throughout the Traders’ Cave (which is less than 50 m wide and largely open on the west side) and within the outer 100 m of the Gan Kira passage.

**Fig. 3:**
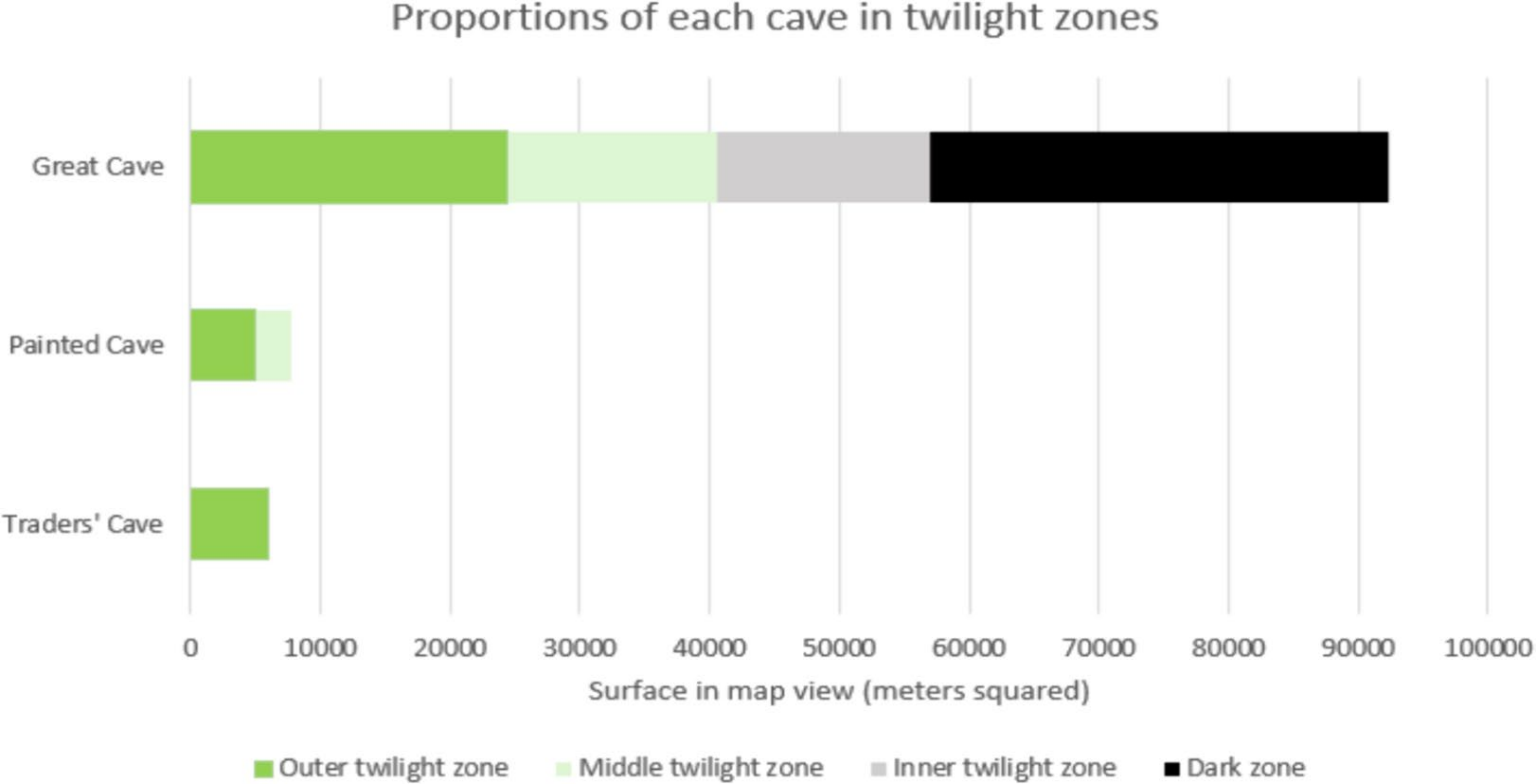
Proportions of caves in twilight and dark zones. Note: for the Great Cave, the Lobang Bulan passage and the Lobang Tahi Menimbun passages have not been included as they were not surveyed

Using the light survey data and mapping information, the extent of the twilight zone has been estimated, considering three twilight sub-zones: an outer twilight zone (within 50 m of an entrance), a middle twilight zone (50 to 100 m of an entrance), and an inner twilight zone (100 to 200 m from an entrance) beyond which is the dark zone (over 200 m from nearest entrance and light levels below detection limit). Figure 3 shows the proportions of each of these zones in the three caves based on the surface areas in the map view.

### Gamma radiation

**Fig. 4:**
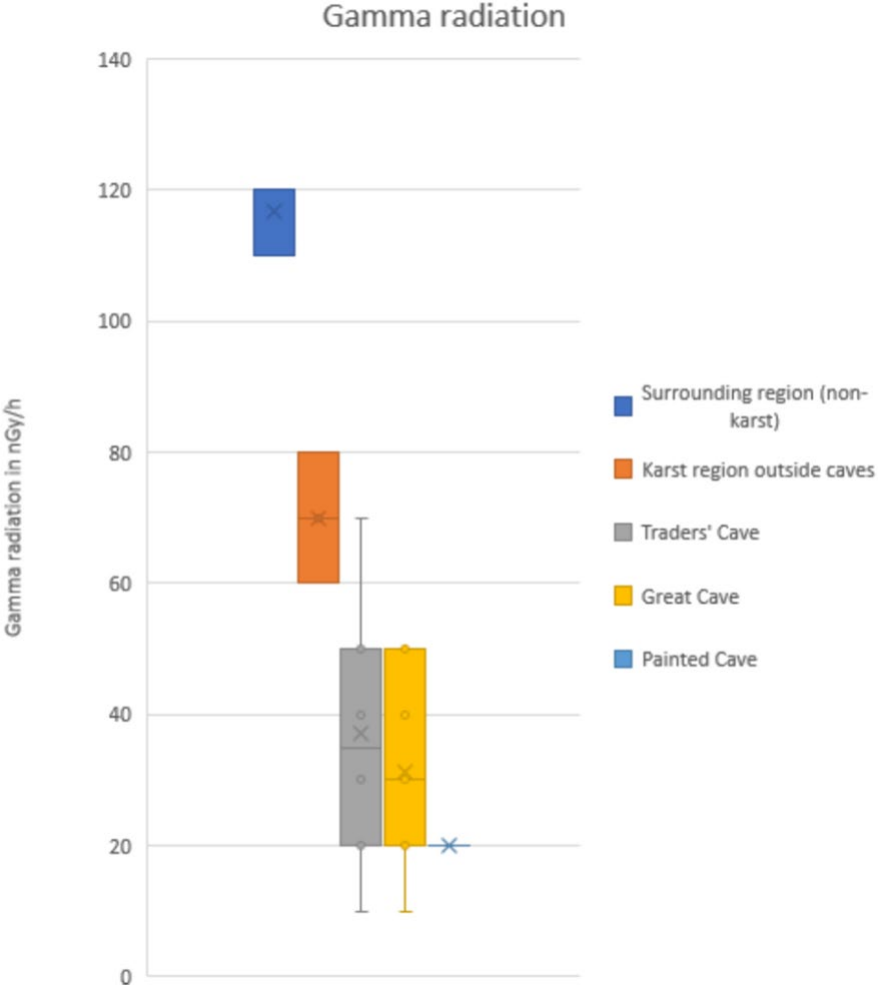
Box and whisker plot of gamma radiation values showing lower values in the caves compared to karst region outside caves and non-karst surrounding country rock

A total of 32 gamma readings were taken, ten of which were in TC, 15 in GC, and one in PC. Six readings were also taken outside the caves including three in the vicinity of the karst towers and alluvial plain and three within a 10 km radius of Niah but not on carbonate rocks. The results are summarized in Fig. 4.

The background terrestrial gamma radiation dose, on fine-grained siliciclastic country rock surrounding the karst region averages 117 nGy/h. Within the karst region but outside the caves, the average is 70 nGy/h. The gamma radiation is significantly lower inside the caves with an average of 31 nGy/h and with some variability from place to place. The lowest values (≤ 20 nGy/h) were observed in the central zone of GC, in the Boulder Field in TC, and in the PC north entrance.

Although additional gamma measurements would be needed to better understand the variability within the caves the results clearly indicate low to very low gamma radiation from the karstified limestone and cave sediments. Gamma radiation inside the caves is less than half of that outside the caves and about 25% of that in the surrounding non-karst region.

### *Beta* flux

**Fig. 5:**
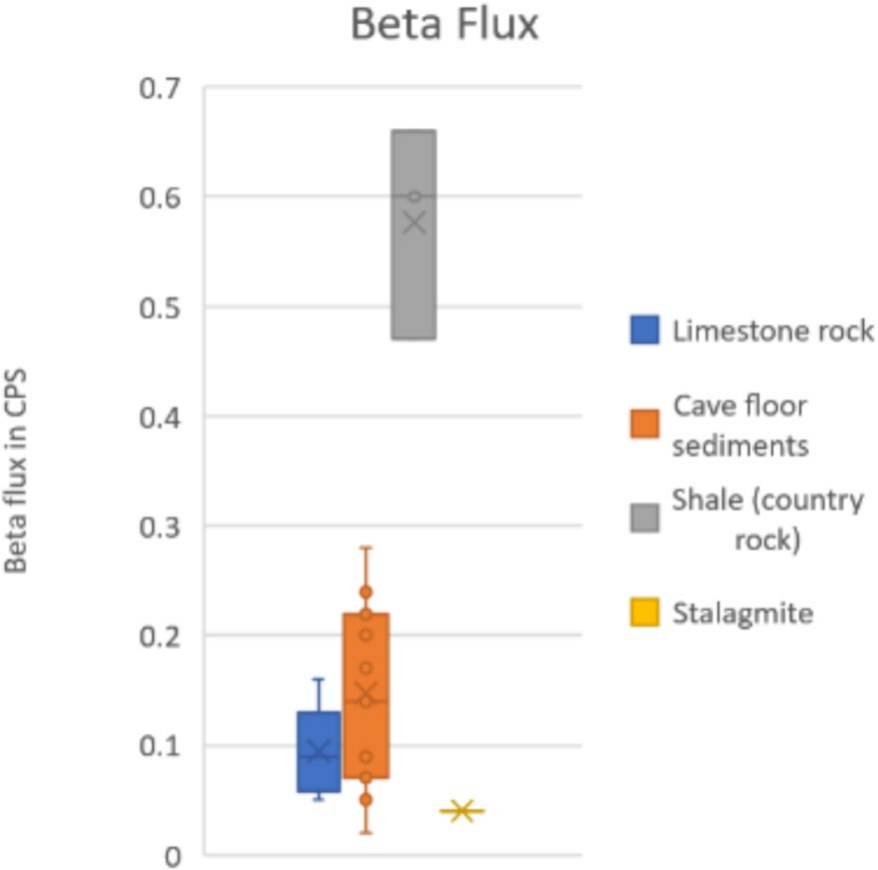
Box and whisker plot of beta flux values showing lower values on stalagmite, limestone rock, and cave floors compared to shale in country rock

Twenty-one measurements of beta flux were made, 11 on cave sediment floor, 6 on limestone rock surfaces, 3 on shales (outside of the karst region), and one on stalagmite. The results are summarized in Fig. 5. The values were consistent and low for the limestone ranging from 0.05 to 0.16 CPS, with an average of 0.095 CPS. They were slightly higher and more variable for the cave sediment floor, which may include blocks of limestone and other materials, ranging from 0.02 to 0.28 CPS, with an average of 0.15 CPS. Significantly higher values were observed outside the karst area on shales, with an average of 0.58 CPS. The beta flux on the stalagmite surface was also low at 0.04 CPS.

### Hydrochemistry

#### In situ* parameters*

**Fig. 6:**
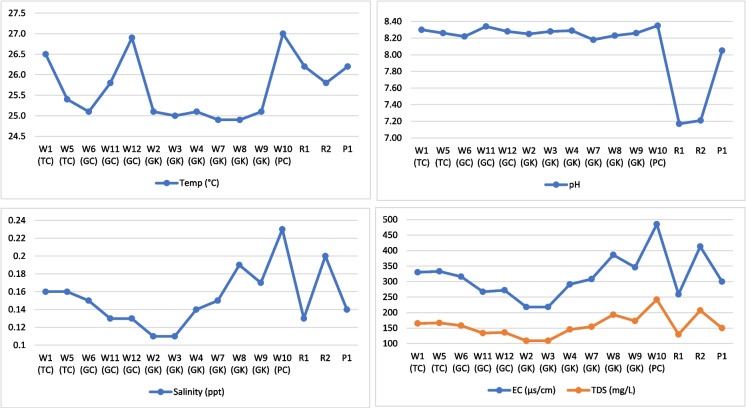
Physical parameters in cave drip waters (W) and surface waters (R and P). Samples marked TC are from Traders Cave, GC from Great Cave, GK from Gan Kira passage, and PC from Painted Cave. Locations for sample numbers W1 to P1 are shown on the map (Fig. 1)

Table 2 shows the elemental concentrations including isotopes in drip water and surface water samples with the descriptive statistical values. In the drip water, the highest temperatures were observed in PC followed by TC, GC, and GK (Fig. 6). For the surface water, the highest temperatures were observed in pooling water in TC (P1). For drip water pH, the highest values were noted in PC followed by TC, GC, and GK. The pH value in PC was the same as the mean value in other caves. For the surface water, the highest pH was observed in P1. The highest EC in drip water was observed in PC followed by GK, TC, and GC. For surface water, the highest EC was noted in streams, particularly in streams to the north of TC (R2). TDS in drip water and surface water follow the same trend as EC. The highest salinity in drip water was observed in PC followed by GK, TC, and GC. For surface water, R2 showed the highest salinity. Based on semi-quantitative observation at the time of sampling (25–27 March 2019), the drip rate was highest in GK, followed by GC, TC, and PC.

**Table 2:** Physicochemical concentrations and statistical values of in situ parameters, major ions, and isotopes in 12 drip water samples and 3 surface water samples

Location	Temperature (°C)	pH	EC (µs/cm)	TDS (mg/L)	Salinity (ppt)	Ca^2+^ (mg/L)	Mg^2+^ (mg/L)	Na^+^ (mg/L)	K^+^ (mg/L)	HCO_3_^−^ (mg/L)	Cl^−^ (mg/L)	SO_4_^2−^ (mg/L)	NO_3_^−^ (mg/L)	NO_2_^−^ (mg/L)	δ^18^O (‰)	δ^2^H (‰)	d-excess
W1 (TC)	26.5	8.30	330	164.8	0.16	410.2	17.8	4.933	6.741	183	212.7	2	2.41	0.55			
W5 (TC)	25.4	8.26	333	166.6	0.16	109	6.9	0.601	5.346	170.8	141.8	3	4.77	1.42	− 3.34	− 16.18	10.54
W6 (GC)	25.1	8.22	316	157.9	0.15	87.5	25.5	0.879	4.213	136	194.9	2	9.13	2.23	− 4.56	− 25.38	11.1
W11 (GC)	25.8	8.34	267	133.7	0.13	61.8	10.2	BDL	5.374	125	124	1	18.08	4.8	− 2.91	− 14.21	9.07
W12 (GC)	26.9	8.28	272	135.8	0.13	47.2	6.3	1.914	4.557	126	159	1	9.03	2.06			
W2 (GK)	25.1	8.25	218	109.0	0.11	68.95	9.4	0.311	3.677	146	106.35	BDL	3.05	0.69			
W3 (GK)	25.0	8.28	218	109.0	0.11	28.5	12.8	2.399	3.733	134.2	141.8	2	4.95	1.12			
W4 (GK)	25.1	8.29	291	145.5	0.14	56.5	13.1	0.831	3.74	134	141	3	9.78	2.2	− 4.1	− 22.12	10.68
W7 (GK)	24.9	8.18	308	154.2	0.15	79.8	12.3	0.122	6.428	109.8	159.5	3	9.67	2.2	− 6.12	− 36.51	12.45
W8 (GK)	24.9	8.23	386	193.2	0.19	120.7	18.4	2.145	10.14	97.6	177.2	2	11.6	6.6			
W9 (GK)	25.1	8.26	346	172.9	0.17	97.6	21.1	1.05	5.3	122	159	1	12.4	8.8	− 6.41	− 38.92	12.36
W10 (PC)	27.0	8.35	485	242.0	0.23	55	49.8	1.849	6.826	146.4	195	6	20.2	14.12	− 4.86	− 27.76	11.12
*Minimum*	*24.9*	*8.2*	*218.0*	*109.0*	*0.1*	*28.5*	*6.3*	*BDL*	*3.7*	*97.6*	*106.4*	*BDL*	*2.4*	*0.6*	* − 6.4*	* − 38.9*	*9.1*
*Maximum*	*27.0*	*8.4*	*485.0*	*242.0*	*0.2*	*410.2*	*49.8*	*4.9*	*10.1*	*183.0*	*212.7*	*6.0*	*20.2*	*14.1*	* − 2.9*	* − 14.2*	*12.5*
*Mean*	*25.6*	*8.3*	*314.2*	*157.1*	*0.2*	*101.9*	*17.0*	*1.4*	*5.5*	*135.9*	*159.4*	*2.2*	*9.6*	*3.9*	* − 4.6*	* − 25.9*	*11.0*
*Std. Dev*	*0.8*	*0.0*	*73.3*	*36.5*	*0*	*100.7*	*11.8*	*1.4*	*1.9*	*23.8*	*31.2*	*1.5*	*5.5*	*4.1*	*1.3*	*9.4*	*1.2*
R1	26.2	7.17	259	129.5	0.13	62.05	32.4	3.745	4.567	134	159	12	1.81	0.42			
R2	25.8	7.21	413	207.0	0.2	392.6	68	4.91	4.863	134.6	141.8	5	18.7	10.8			
P1	26.2	8.05	300	150.0	0.14	29.3	19.1	1.524	2.88	110	159.5	BDL	4.61	1.15			
*Minimum*	*25.8*	*7.2*	*259.0*	*129.5*	*0.1*	*29.3*	*19.1*	*1.5*	*2.9*	*110.0*	*141.8*	*BDL*	*1.8*	*0.4*			
*Maximum*	*26.2*	*8.1*	*413.0*	*207.0*	*0.2*	*392.6*	*68.0*	*4.9*	*4.9*	*134.6*	*159.5*	*12.0*	*18.7*	*10.8*			
*Mean*	*26.1*	*7.5*	*324.0*	*162.2*	*0.2*	*161.3*	*39.8*	*3.4*	*4.1*	*126.2*	*153.4*	*5.7*	*8.4*	*4.1*			
*Std. Dev*	*0.2*	*0.5*	*79.8*	*40.2*	*0*	*201.0*	*25.3*	*1.7*	*1.1*	*14.0*	*10.1*	*6.0*	*9.1*	*5.8*			

Samples marked TC are from Traders Cave, GC from Great Cave, GK from Gan Kira passage, and PC from Painted Cave; R1, R2, and P1 from surface water. *BDL*, below detection limit

#### Major ions

**Fig. 7:**
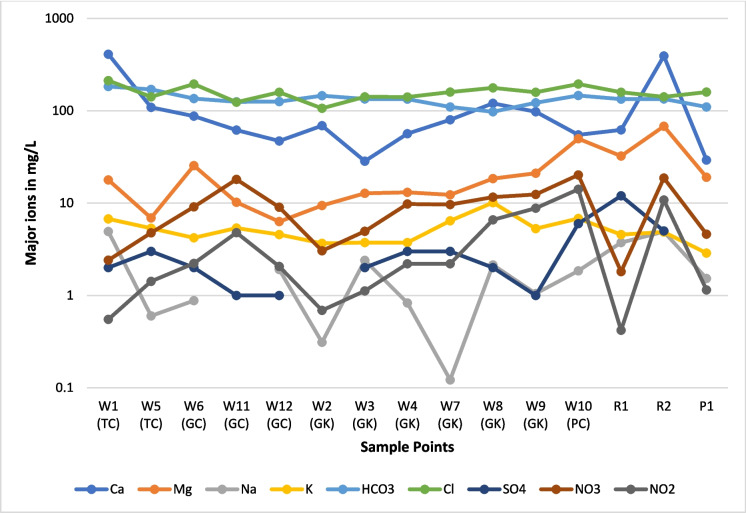
Concentration of major ions in cave drip waters (W) and surface waters (R and P). Samples marked TC are from Traders Cave, GC from Great Cave, GK from Gan Kira passage, and PC from Painted Cave. Locations for sample numbers W1 to P1 are shown on the map (Fig. 1)

Major ion concentrations are shown in Fig. 7. In drip water, Ca^2+^ is the dominant cation, and the highest Ca^2+^ values were observed in TC, followed by GK, GC, and PC. For Mg^2+^, the highest values were noted in PC followed by GC, GK, and TC. The highest values of K^+^ were observed in PC followed by TC, GK, and GC. The highest values of Na^+^ were observed in TC, followed by PC, GK, and GC. For surface water, the highest cation values were observed in stream water, particularly in R2. For anions, Cl^−^ is the dominant ion, and the highest values were observed in PC followed by TC, GC, and GK. For HCO_3_^−^, the highest values were observed in TC followed by PC, GK, and GC. The highest values of NO_3_^−^ and NO_2_^−^ were observed in PC, followed by GC, GK, and TC. For SO_4_^2−^, the highest values were noted in PC, followed by TC, GK, and GC. For surface water, as is the case for cations, the highest anion values were observed in stream water, particularly in R2.

## Discussion

### Light, temperature, and humidity

Light, temperature, and humidity in caves depend on a number of interrelated factors such as mean conditions outside the cave, air and water flow, and cave configuration, particularly the location and orientation of entrances (De Freitas et al., 1982; Hasegawa et al., 2018; Mejía-Ortíz et al., 2021).

The Niah Caves have numerous large openings on different sides of the karst tower as well as several ceiling skylights; hence, there is an extensive twilight zone in which both light, temperature, and humidity are expected to vary quite significantly both diurnally and seasonally. In fact, it is not known if any parts of the Niah cave(s) have constant temperature or humidity which might be expected in a cave with less communication with the outside (Mejía-Ortíz et al., 2021). The results of this study indicate that almost half of the area in the caves is within 100 m of an entrance, where light and other external influences are significant and where light is sufficient for cyanobacteria and plant growth. Maximum light intensity exceeds 2000 lx at the brightest times of the day in the PC entrance (Dodge-Wan et al., 2012). Similarly, many parts of TC and GC (West Mouth) are in direct sunlight during part of the day. Only 37% of the GC areas are in the dark zone, and none of TC and PC is dark, except for very limited small restricted areas and narrow spaces.

This limited study shows spatial variations in air temperature between 25.9 and 30.5 °C. Humidity shows an inverse relationship with temperature. Six readings from the center of GC towards the southeast along the GK passage and to the Lobang Gan Kira entrance showed temperature decreasing by 2° over 400 m distance towards that entrance and with an intermediate value outside in the forest.

Humidity in caves is generally higher than mean values outside at the land surface, and often close to saturation (Mejía-Ortíz et al., 2021). At Niah, the majority of the relative humidity readings during the day were between 75 and 80%, which was actually lower than outside at the same time. McClure et al. (1967) report high seasonal variations in humidity in the Dark Cave (Batu Caves, Kuala Lumpur, Malaysia), a smaller cave with significant but less entrances than Niah. Further research is required to establish the full temperature and humidity ranges within the caves and to fully understand their variations. Short-term observational data is insufficient to fully understand the complexity of temperature and humidity variations in complex cave passages with many communications between the cave and the outside, such as ceiling skylights and large entrances. It would be desirable also to gain an understanding of how the underground conditions vary in response to variations outside of the caves.

### Wind

Cave wind can be expected to vary diurnally with changes in the outside temperature which causes differences in the density gradient between inside and outside environments (De Freitas et al., 1982). Density gradients will lead to pressure differences between entrances, the inside and the outside (Luetscher & Jeannin, 2004). Wind velocity in caves is affected by these gradients and also by passage size, shape, and configuration, with some areas being well-ventilated and others possibly stagnant in dead-end passages (De Freitas et al., 1982).

There is little available data on cave wind from other tropical caves for comparison. Hasegawa et al. (2018) report wind speeds of zero to > 0.4 m/s in Petruk Cave (Java, Indonesia), a 350-m-long cave with an underground river and much smaller passages than those of Niah. The highest wind velocity values recorded in this study at PC and GK passages are much greater and also measured in significantly larger passages.

The preliminary air flow rates obtained at Niah (122 m^3^/s at GK and 530 m^3^/s at PC) are one to two orders of magnitude greater than those reported under different climatic conditions at Glowworm Cave in New Zealand (De Freitas et al., 1982) and in alpine caves (Luetscher & Jeannin, 2004).

At Niah, it would be necessary to monitor air temperature, humidity, and wind over several days or seasons to better study those variations. It is not known to what extent wind outside the caves is also an influencing factor.

### Radiation

Gillmore et al. (2005) conducted a study of radon concentrations in and around the archeological trench sites in the Great Cave West Mouth which may be poorly ventilated. They concluded that the radon levels in Great Cave were very low compared to world standards.

This study of gamma radiation showed low values in the karst (70 nGy/h) which are significantly lower than in the surrounding area of fine-grained clastic sediments, where an average TGRD of 117 nGy/h was recorded. An even lower average value of 31 nGy/h was measured inside the caves. The world median TGRD is 59 nGy/h, and the range in Malaysia is 55 to 130 nGy/h (UNSCEAR, 2000). The average outdoors on the Curtin campus site on a clastic sedimentary rock with Quaternary cover is reported to be 81 nGy/h (Dodge-Wan & Prasanna, 2021).

Beta flux was also low on limestone surfaces and approximately six times higher on clastic rocks outcropping outside of the karst region.

The findings of this study therefore indicate lower than regional average TGRD in the karst and caves, hence no cause for concern to visitors. In fact, from a radiological point of view, the well-ventilated caves are safer than the outside environment.

### Hydrochemistry

There is not much variation in temperature, pH, and salinity values of drip water in all the caves. However, EC and TDS values varied significantly due to the spatial variation of ionic concentrations in the drip water. Ca^2+^ and Cl^−^ are the most dominant cation and anion in the drip waters. The major ions in drip water are Ca^2+^  > Mg^2+^  > K^+^  > Na^+^ and Cl^−^  > HCO_3_^−^  > NO_3_^−^  > NO_2_^−^  > SO_4_^2−^ in decreasing order of concentration, irrespective of the sample point. However, in surface waters, the following decreasing order of major ions was observed Ca^2+^  > Mg^2+^  > Na^+^  = K^+^ and Cl^−^ > HCO_3_^−^  > NO_3_^−^  > SO_4_^2−^  > NO_2_^−^. In terms of ionic concentration, drip water in PC has the higher ionic concentration, followed by that of TC, GK, and GC. However, only one sample was collected at the entrance of the PC.

### Geochemical processes

**Fig. 8:**
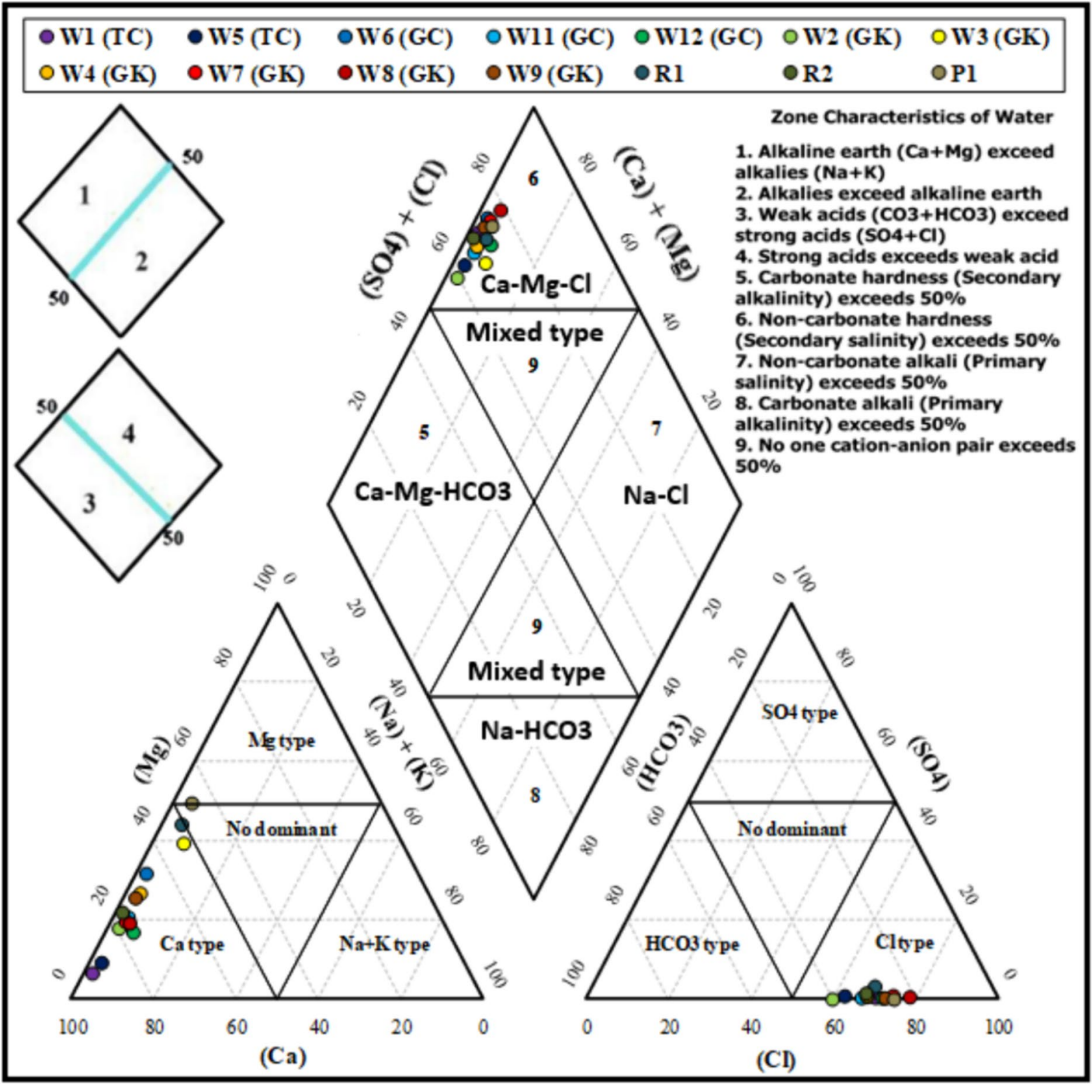
Piper plot to show the water types in the caves and in three surface water samples. Samples marked TC are from Traders Cave, GC from Great Cave, GK from Gan Kira passage, and PC from Painted Cave. Locations for sample numbers W1 to P1 are shown on the map (Fig. 1)

**Fig. 9:**
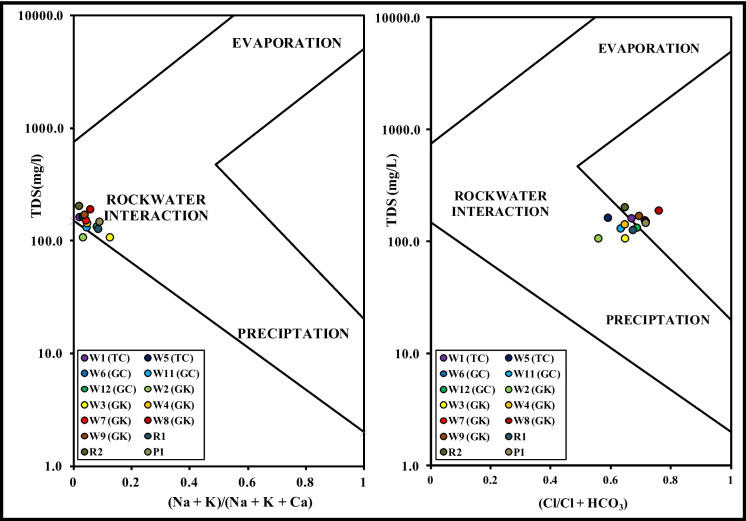
Gibbs plot to identify the major geochemical processes in the cave waters and three surface water samples. Samples marked TC are from Traders Cave, GC from Great Cave, GK from Gan Kira passage, and PC from Painted Cave. Locations for sample numbers W1 to P1 are shown on the map (Fig. 1)

The data on percentages of major ions was used to visualize the geochemistry on the Piper diagram (Piper, 1944) in order to identify the water types and the related sources (Fiona Bassy et al., 2024; Parvin Raj & Prasanna, 2023; Stephan et al., 2022). On the Piper diagram (Fig. 8), all the waters tested fell in the Ca–Mg–Cl field, with variable proportions of calcium and magnesium. This indicates the predominance of weathering and dissolution of carbonate minerals through ion exchange reactions between rock/sediments and water (Prasanna et al., 2014; Zhang et al., 2020). This inference was also supported by the Gibbs plot (Gibbs, 1970), where all the samples fell in the rock-water interaction zone (Fig. 9).

**Fig. 10:**
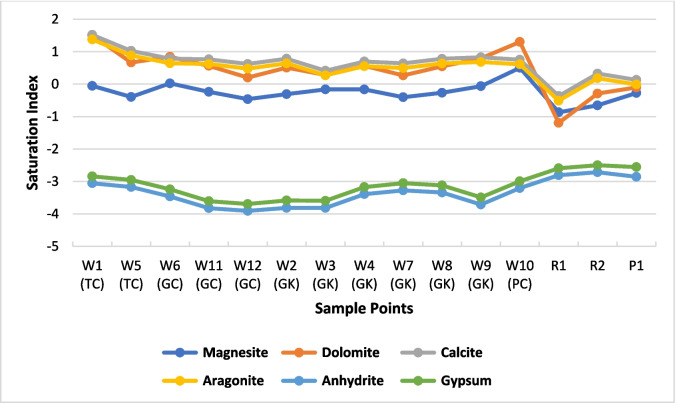
Saturation index for carbonate and sulfate minerals in the drip water and three surface water samples

The calculated saturation index (SI) values are shown in Fig. 10. They indicate the trend of water and mineral chemical equilibrium and water–rock interaction (Chidambaram et al., 2012; Rakesh et al., 2023; Zhang et al., 2020). For all water samples, the SI indices show under-saturation for anhydrite and gypsum, which indicates the potential for continuous dissolution of sulfate minerals. Whereas, for calcite, aragonite, and dolomite, the indices show over-saturation which indicates the potential for precipitation of carbonate minerals. It is also suggested that the addition of Ca^2+^ from the weathering of anhydrite and gypsum could lead to the over-saturation of carbonate minerals, thus inhibiting the weathering of calcite and dolomite (Zhang et al., 2020). The SI of magnesite was found to be close to the equilibrium state.

### Isotopic signatures

**Fig. 11:**
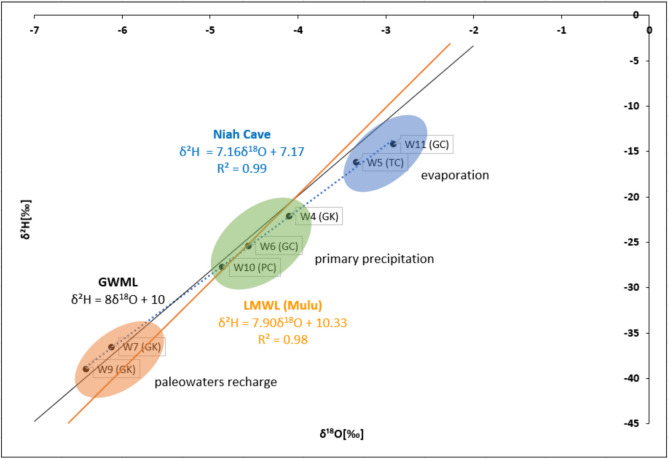
*δ*^18^O-*δ*^2^H diagram to depict the sources and the processes of drip water from the Niah cave complex. Samples GK are from Gan Kira, PC from Painted Cave, GC from Great Cave, and TC from Traders’ Cave

The isotopic values of Niah Cave drip water were plotted on the *δ*^18^O-*δ*^2^H diagram (Fig. 11). Mulu’s atmospheric precipitation line (Ninu Krishnan et al., 2022) was used as the local meteoric water line (LMWL), as Mulu is relatively close to the current study location. The global meteoric water line (GMWL) was also plotted in the diagram (Craig, 1961). δ^18^O ranges from − 6.41 to − 2.91‰ with a mean of − 4.61‰, whereas δ^2^H ranges from − 38.92 to − 14.21‰ with a mean of − 25.87‰. The heavier isotopes were observed in TC and GC drip waters, and the lighter isotopes were observed in GK and PC. The d-excess value ranges from 9.07 to 12.45‰ with a mean of 11.05‰. The Niah Cave drip water line showed a strong positive correlation (R2 = 0.99) between *δ*^18^O and *δ*^2^H with the following equation:

$${\delta }^{2}\text{H}=7.16{\delta }^{18}\text{O}+7.17\cdots {R}^{2}=0.99$$

**Fig. 12:**
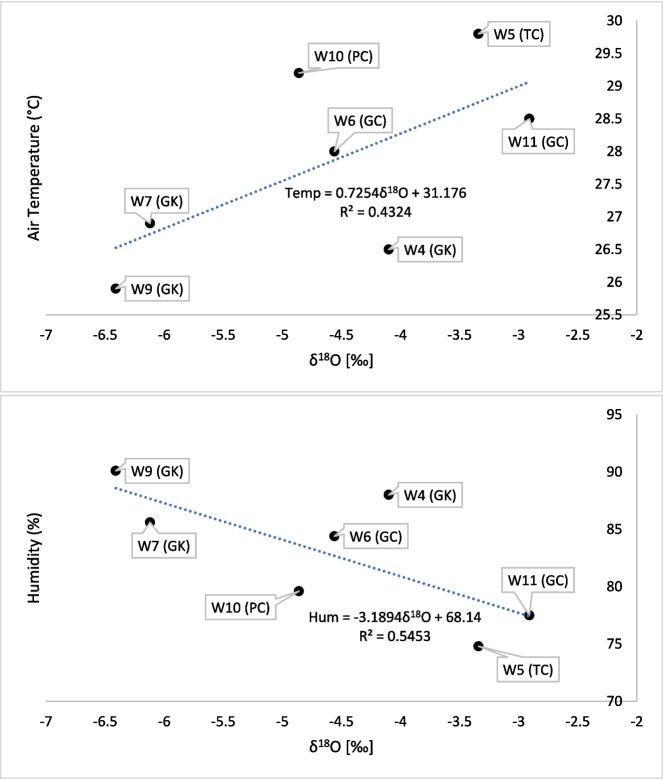
Correlation plots of cave air temperature and humidity with δ^18^O

The similar slope values between Niah Cave drip water and LMWL indicate a common moisture source in these two locations, which is the formation of rain in the equatorial tropical region (Dansgard, 1964; Fiona Bassy et al., 2024; Ninu Krishnan et al., 2022). However, the difference in the d-intercepts indicates the possibility of re-evaporation owing to the variation in humidity, temperature, and changing condition of moisture source in these locations (Rozanski et al., 1982). The positive correlation between air temperature and δ^18^O and the inverse correlation between humidity and δ^18^O clearly indicate the influence of the cave environment on the δ^18^O values in drip water (Fig. 12).

**Fig. 13:**
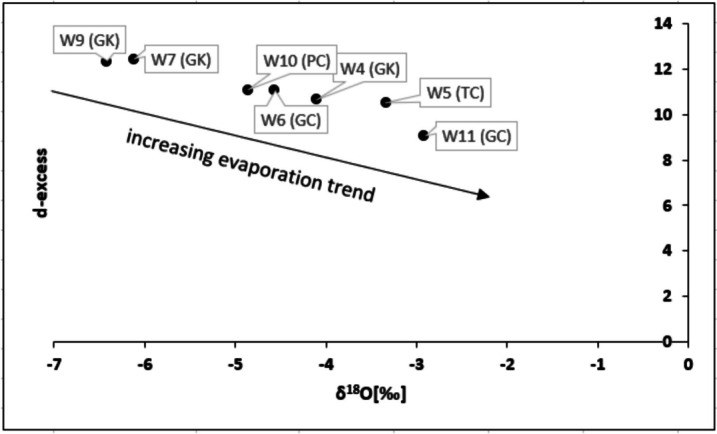
d-excess-*δ*^18^O diagram for evaporation process in the drip water from Niah cave complex. Samples GK are from Gan Kira, PC from Painted Cave, GC from Great Cave, and TC from Traders’ Cave

In Fig. 11, three clusters of samples have been identified based on the variation of isotopic values in the drip water. Samples, W4, W6, and W10, fall close to the LMWL and GMWL indicating recharge from direct precipitation (Fiona Bassy et al., 2024; Zhang et al., 2021). However, samples, W5 and W11, deviate from LMWL which indicates an evaporation process (Prasanna et al., 2010). These sample sites are at the cave entrance, where the distance between the high cave ceiling where the drip forms and the sample point close to the cave floor is significant. At the time of sampling, the drip rate in these locations was slow to medium based on semi-quantitative observation. Hence, the drip waters may have been subjected to secondary evaporation during the fall of the waterdrop (Thivya et al., 2016; Xia et al., 2021). Comparatively lower humidity (avg. 76.15%) and higher air temperature (avg. 29 °C) at these locations also support the evaporation process which led to enriched δ^18^O isotopic values. Samples, W7 and W9, fall closer to the GMWL in Fig. 11 but deviate towards the left from the LMWL, which indicates paleo-water recharge (Clark & Fritz, 1997). Both samples were collected from the narrow passage of GK where there is a thick overburden ceiling. The previous episodes of rainwaters in the cracks and fissures of the rock above could be remobilized by more recent rainwater recharge and hence reflects a cumulative mixture of past rainwater compositions (Cruz et al., 2005; Prasanna et al., 2010).

d-excess-*δ*^18^O diagram (Fig. 13) shows a trend of increasing evaporation from the south to the north of Niah Cave. Broad cave openings with relatively lower humidity and high air temperature in TC and GC support the high evaporation in those locations with enriched isotopic values in the drip waters.

### Factor analysis

Five factors were extracted through principal component analysis of the physicochemical parameters of drip waters, three surface water samples, and the respective cave environmental parameters including radiation data. Five factors were extracted that explain 88.69% of the total variance (Table 3). Factor 1 is loaded with EC, TDS, salinity, Ca^2+^, Mg^2+^, NO_3_^−^ and NO_2_^−^, and explains 28.95% of the total variance. Of these parameters, Ca^2+^ and Mg^2+^ reflect weathering and dissolution of carbonate minerals (Ninu Krishnan et al., 2020) whereas NO_3_^−^ and NO_2_^−^ are derived from the leaching of biogenic sediments and guano that increases the ionic concentration in the drip waters at W11, W9, and R2 (Khanaqa & Al-Manmi, 2011). The presence of large amounts of calcite in the bedrock of Niah Cave is also confirmed by the above observation (Prasanna et al., 2014). The negative loading of pH with other ions also supports the dissolution of carbonate minerals (Parvin Raj & Prasanna, 2023). Factor 2 is loaded with EC, TDS, salinity, K^+^, and Cl^−^ and explains 19.13% of the total variance. K^+^ and Cl^−^ are derived from the leaching of clay minerals and soils coated on the cave roof, which increases the ionic concentration at the sample sites, W1, W5, W6, W7, W8, and W9. Factor 3 is loaded with gamma radiation, Mg^2+^ and SO_4_^2−^, and explains 16.41% of the total variance. Mg^2+^ indicates the excess contribution to the formation of dolomite in association with Ca^2+^ (Prasanna et al., 2014), whereas SO_4_^2−^ is derived from the leaching of clay/soil (Mayer, 1999). Interestingly, W3 and W7 in GK have the highest gamma radiation. W3 is in a relatively narrow part of the passage where there is less ventilation (BDL for wind velocity). Factor 4 is loaded with Ca^2+^, Na^+^, and HCO_3_^−^ and explains 13.43% of the total variance. Ca^2+^ and HCO_3_^−^ are derived from the dissolution of calcite through water–rock interaction by recharge waters (Freeze & Cherry, 1979), whereas Na^+^ is probably derived from ion exchange in soils and clastic rocks (Mayer, 1999). This factor was observed in W1, W5, W6, W2, W3, R2, and P1. Factor 5 is loaded with temperature, light, and wind and explains 10.76% of the total variance. These environmental parameters have limited influence on the drip water and surface water chemistry. This factor was observed in W1, W11, and W12, which are from the large entrance at the West Mouth of TC and GC to receive more light and wind and have high temperatures.

**Table 3:** Factor analysis with the factor scores for the analyzed physicochemical and environmental data

Parameters	Component				
	1	2	3	4	5
Temperature	0.069	− 0.049	0.108	0.278	**0.901**
pH	− 0.451	0.020	− 0.858	− 0.062	− 0.115
EC	**0.604**	**0.740**	0.025	− 0.032	− 0.086
TDS	**0.606**	**0.738**	0.027	− 0.034	− 0.088
Sal	**0.605**	**0.751**	0.060	− 0.049	− 0.105
Air temperature	− 0.931	− 0.014	− 0.303	− 0.036	0.101
Humidity	− 0.933	0.060	− 0.160	− 0.195	− 0.087
Light	− 0.294	− 0.025	0.427	− 0.050	**0.781**
Wind	0.049	0.100	− 0.429	− 0.653	**0.580**
Gamma radiation	0.114	− 0.484	**0.724**	− 0.118	0.142
Ca^2+^	**0.621**	0.423	0.007	**0.589**	0.120
Mg^2+^	**0.786**	0.161	**0.500**	0.141	− 0.067
Na^+^	0.389	0.314	0.445	**0.509**	0.347
K^+^	0.005	**0.818**	− 0.076	− 0.241	− 0.003
HCO3^−^	− 0.031	− 0.074	− 0.108	**0.839**	0.160
Cl^−^	− 0.183	**0.794**	− 0.021	0.363	0.153
SO_4_^2−^	− 0.008	0.105	**0.905**	0.061	0.116
NO3^−^	**0.721**	0.116	− 0.262	− 0.539	− 0.019
NO2^−^	**0.803**	0.309	− 0.061	− 0.413	− 0.086
Total	5.501	3.636	3.118	2.552	2.045
% of variance	28.951	19.134	16.411	13.432	10.763
Cumulative %	28.951	48.085	64.496	77.928	88.690
Factor scores					
Sample location	FS1	FS2	FS3	FS4	FS5
W1 (TC)	− 0.22965	**1.35775**	− 0.80249	**2.5871**	**0.89468**
W5 (TC)	− 0.35533	**0.09412**	− 0.02262	**0.57006**	− 0.11752
W6 (GC)	− 0.33139	**0.60331**	− 0.51175	**0.52208**	− 0.82711
W11 (GC)	**0.42931**	− 0.7798	− 1.34305	− 1.18761	**0.73679**
W12 (GC)	− 0.26633	− 0.40784	− 0.46016	− 0.72148	**2.65665**
W2 (GK)	− 0.31823	− 1.71147	− 0.40751	**0.43064**	− 0.76597
W3 (GK)	− 0.50956	− 1.1579	**0.0495**	**0.35625**	− 0.68576
W4 (GK)	− 0.19715	− 0.47337	− 0.13202	− 0.01188	− 0.83575
W7 (GK)	− 0.48045	**0.33355**	**0.36492**	− 0.66838	− 1.08534
W8 (GK)	− 0.19994	**2.1842**	− 0.00099	− 1.42249	− 0.45552
W9 (GK)	**0.34657**	**0.60374**	− 0.63197	− 0.90933	− 0.25513
R1	− 0.96192	− 0.0658	**2.90324**	− 0.04493	**0.83076**
R2	**3.27125**	− 0.07111	**0.96431**	**0.39459**	− 0.06342
P1	− 0.19715	− 0.50938	**0.03058**	**0.10536**	− 0.02734

Values shown in bold are statistically significant

## Conclusion

The traverse line survey results, obtained with modern instruments, confirm the quality of previous mapping by Wilford (1964) and confirm the vast size of cave entrances, the height of ceiling skylights, and provide relative elevation information that was previously lacking. The Gan Kira passage was surveyed at − 13 m below datum G1, which is at a timber walkway near the archeological site in West Mouth. Passage cross-section surveys combined with wind speed measurements at the time of the survey in March 2019 allow airflow rates to be established. The flow was 122 m^3^/s within 100 m of Lobang Gan Kira (Gan Kira entrance) flowing to the southeast. In the “light through” Painted Cave airflow was also to the southeast at 530 m^3^/s. Both flow rates obtained in this study are significant, indicating substantial air flow through the cave system. Depending on passage configuration and size, in other parts of the cave, airflow rates were below the detection limit, emphasizing the variability of the cave environment in relationship to specific factors.

Many of the day-time air temperature measurements made in the caves exceed 29 °C with a high value of 30.5 °C recorded in West Mouth. The lowest values in the cave at the time of the survey were 25.9 °C in the Gan Kira passage. Humidity in the caves varied from 72% to over 90%, with high values (over 91%) in adjacent swamp forests. An inverse correlation between temperature and humidity was noted.

Light intensity survey indicates strong spatial variations depending on obstacles and distance from the nearest entrance. All of the Traders’ Cave and Painted Cave are within twilight zones. More than half of the surface area of Great Cave is also within the twilight zone, although the survey did not include Lobang Bulan and Lobang Tahi Menimbun which are expected to contain dark zone passages. The results are limited to day-time values; hence, they are expected to only show part of the full diurnal variability of the light, airflow, temperature, and humidity that may occur.

Background terrestrial gamma radiation was found to be very low in the caves, with an average of 31 nGy/h. In the karst region outside the caves, the average is also low at 70 nGy/h. Higher values were noted in the surrounding non-karst region, with predominantly fine clastic rock types where the average is 117 nGy/h. Beta flux values show a similar pattern, i.e., very low on stalagmite, cave floor, and limestone and up to six times higher on shale country rock.

Physical parameters measured in situ in the drip water do not show major variations between the sample locations in the various parts of the three caves that were studied. Ca^2+^, Mg^2+^, Cl^−^, and HCO_3_^−^ are the dominant ions in the drip water which confirms the carbonate mineral–water equilibrium. Ca-Mg-SO_4_ is the dominant water type an indication of the dissolution of carbonate and sulfate minerals. Water–rock interaction is the main process which controls the chemistry of the drip water. The results show under-saturation of sulfate minerals which indicates dissolution. They show over-saturation of carbonate minerals which confirms precipitation. Air temperature and humidity both influence the δ^18^O values in drip water. Based on the isotopic signatures, drip waters originate from direct precipitation as might be expected, with some paleo-water recharge from water present in cracks and fissures of the limestone mass. They also show secondary evaporation in some cases. The similar slope values observed between Niah Cave drip water and LMWL at Mulu indicate a common moisture source in the two locations, which is the formation of rainfall in this equatorial tropical region. Cave entrance drip water is also subjected to evaporation depending on humidity and air temperature. d-excess values indicate an increasing evaporation trend from the south to north within the Niah cave. Factor analysis reveals weathering and dissolution of carbonates, and leaching from the biogenic sediments/soils/clay in the rock surface is the main controlling factor for the hydrochemistry of drip waters in Niah Cave.

Future research should aim to establish long-term variations of some of the parameters established at the baseline level in this study: air temperature, humidity, and wind speed should be monitored in a selected number of locations in a semi-continuous manner to detect diurnal and seasonal variations. Similarly, increasing the parameters, number of locations, and seasonal sampling could enhance the interpretation of the hydrochemistry of cave waters. The ongoing impact of climate change and the expected increase in visitor numbers following the inscription of Niah National Park’s Cave Complex on the UNESCO World Heritage List (UNESCO, 2024) warrants long-term environmental monitoring.

## Data Availability

The datasets used and analysed during the current study are available from the corresponding author on reasonable request.

## Abbreviations

BDL: Below detection limit
CPS: Counts per second
EC: Electrical conductivity
GC: Great Cave
GK: Gan Kira passage (in Great Cave)
GMWL: Global meteoric water line
LMWL: Local meteoric water line
PC: Painted Cave
RH: Relative humidity
SI: Saturation index
TGRD: Terrestrial gamma radiation dose rate
TDS: Total dissolved solids
TC: Traders’ Cave
